# HbA1c screening for diabetes mellitus and to evaluate diabetic control in people of African ancestry with HIV in South London

**DOI:** 10.1177/09564624231162163

**Published:** 2023-03-15

**Authors:** Laura Cechin, Lucy Campbell, Amelia Oliveira, Louise M Goff, Frank A Post

**Affiliations:** 1 8948 King’s College Hospital NHS Foundation Trust, London, UK; 2 4616 King’s College London, London, UK

**Keywords:** HbA1c, HIV, black ethnicity, diabetes

## Abstract

We evaluated glycaemic status in 948 Black adults with HIV and report a high prevalence of dysglycaemia (37.2%). HbA1c testing identified 38 (4.0%) individuals not previously known to have diabetes mellitus (DM) and showed suboptimal or poor glycaemic control in more than half of those with a prior DM diagnosis despite high levels of HIV control.

## Introduction

Type 2 diabetes mellitus (T2DM) is an age-related comorbidity with major adverse effects on the cardiovascular system, kidneys, liver, and peripheral nervous system. Black African and African-Caribbean people are up to five times more likely than white people to develop T2DM and have an earlier onset of diabetes and its complications.^1^ The prevalence of T2DM increases with age and body mass index (BMI); men and Black populations with HIV are disproportionally affected.^2^ As HIV care includes regular follow up and metabolic screening for antiretroviral toxicities and comorbidities, HIV clinics are well placed to detect, and potentially manage T2DM, particularly in individuals who are not accessing population screening programs offered in general practice. The British HIV Association recommends inclusion of HbA1c as part of annual monitoring of people aged 40 or over on antiretroviral therapy (ART),^3^ although this has not been universally implemented.

King’s College Hospital serves an ethnically diverse population with HIV and introduced routine HbA1c testing in November 2020. We performed an audit among Black adults with HIV to assess (1) the coverage of HbA1c testing in routine clinical HIV practice; (2) the extent to which HbA1c testing resulted in new diagnoses of DM; (3) onward referral of those newly diagnosed with DM to diabetic or primary care services; (4) glycaemic control in those with a prior DM diagnosis.

## Methods

We reviewed available HbA1c measurements for participants in the genetic markers of chronic kidney disease in people of African ancestry with HIV (GEN-AFRICA) study^4^ who received HIV care in 2020/2021 at King’s College Hospital, South London, UK. To account for pandemic disruption of HIV care, we included HbA1c measurements performed in 2018/2019, and in January/February 2022, and used the most recent measurement if multiple tests had been performed.

A diagnosis of DM was based on the use of oral hypoglycaemic medications, insulin, or an HbA1c ≥ 48 mmol/mol (≥6.5%); those with an HbA1c of 39–47 mmol/mol (5.7–6.4%) were classified as having prediabetes, and those with HbA1c of ≤38 mmol/mol (5.6%) as having normoglycaemia.^5^ Dysglycaemia was defined by the presence of diabetes or prediabetes. Demographics, HIV parameters and co-morbidities of the audit population were described by glycaemia status and compared using X^2^ for categorical variables, and, based on distribution of the data, Kruskal-Wallis (medians) or ANOVA (means) for continuous data. Statistical analyses performed in STATA v16 (StataCorp, College Station, Tx).

Participants with DM were stratified into those with a prior DM diagnosis and those with a new DM diagnosis. For the latter, we reviewed clinical records to ascertain if they had been notified and/or referred for further confirmatory testing and/or management. Of those with a prior DM diagnosis, we evaluated the proportions that met the National Institute for Clinical Excellence (NICE) target (HbA1c ≤ 7.0%)^6^ and the national audit standard (HbA1c ≤ 7.5%) for glycaemia control,^7^ and described the diabetic medications received by those with HbA1c within and above the HbA1c target. We also reviewed the records for those with poor glycaemia control (failing the diabetes audit standard) for subsequent treatment intensification and improved DM control as well as concomitant blood pressure and HIV control.

## Results

A total of 1,070 GEN-AFRICA participants received HIV care in 2020/2021, of whom 122 (11.4%) did not have an HbA1c result. Individuals without an HbA1c result had similar HIV characteristics as those with HbA1c data but were younger (mean age 45.5 vs 51.0 years) and had a lower mean body mass index (BMI; 27.1 vs 28.8 kg/m^2^), they also had somewhat better kidney function and less often reported hypertension.

Of the 948 (88.6%) with available HbA1c measurements, 457 (48.2%) had normoglycaemia and 491 (51.8%) had dysglycaemia (prediabetes: 353 [37.2%], DM 138 [14.6%], of whom 137 had T2DM). The clinical characteristics of the audit participants, stratified by glycaemia status, are shown in Table 1. As expected, participants with dysglycaemia were older, had higher BMI and lower eGFR, and were more likely to have hypertension. Similar results were obtained when 49 participants with HbA1c measurements in 2018/2019 were excluded: 432/899 (48.1%) had normoglycaemia, and 467 (51.9%) had dysglycaemia (prediabetes: 334 [37.2%], DM 133 [14.8%], of whom 132 had T2DM).

**Table 1.** Participant characteristics.

		Total	Normoglycaemia	Pre-diabetes	Diabetes mellitus	*p*-value
		*N* = 948	*N* = 457	*N* = 353	*N* = 138	
Age, years	Mean (SD)	51.0 (9.7)	48.4 (9.7)	52.4 (8.8)	56.2 (9.1)	<0.001^a^
Sex (female)	*N* (%)	524 (55.3)	258 (56.5)	199 (56.4)	67 (48.6)	0.23^b^
Time since HIV diagnosis, years	Mean (SD)	13.6 (6.1)	13.5 (6.1)	14.1 (5.9)	12.7 (6.7)	0.054^a^
Prior AIDS	*N* (%)	203 (23.1)	99 (23.3)	74 (22.4)	30 (24.0)	0.93^b^
Nadir CD4 (cells/mm^3^)	Median (IQR)	223 (91–358)	229 (95–364)	230 (108–357)	177 (64–357)	0.23^c^
Recent CD4 (cells/mm^3^)	Median (IQR)	573 (390–759)	564 (390–739)	605 (411–779)	533 (377–771)	0.15^c^
On antiretroviral therapy	*N* (%)	939 (99.1)	452 (98.9)	350 (99.2)	137 (99.3)	0.90^b^
HIV RNA undetectable	*N* (%)	878 (94.6)	430 (96.2)	321 (93.0)	128 (93.4)	0.12^b^
HBsAg positive	*N* (%)	62 (6.5)	26 (5.7)	25 (7.1)	11 (8.0)	0.56^b^
HCV Ab positive	*N* (%)	17 (1.8)	9 (2.0)	5 (1.4)	3 (2.2)	0.79^b^
BMI, kg/m^2^	Median (IQR)	28.8 (25.3–32.9)	27.6 (24.0–31.6)	29.4 (26.4–33.3)	30.9 (27.7–35.4)	<0.001^c^
Hypertension	*N* (%)	318 (33.5)	112 (24.5)	117 (33.1)	89 (64.5)	<0.001^b^
Cardiovascular disease	*N* (%)	45 (4.7)	19 (4.2)	17 (4.8)	9 (6.5)	0.52^b^
eGFR (mL/min/1.73 m^2^)	Median (IQR)	100 (83–116)	103 (85–120)	100 (84–115)	86 (70–104)	<0.001^c^

Normoglycaemia, prediabetes and diabetes mellitus are defined by HbA1c < 39 mmol/mol (<5.7%), 39–47 mmol/mol (5.7–6.4%) and ≥48 mmol/mol (≥6.5%) or use of hypoglycaemic medications, respectively. HBsAg = hepatitis B surface antigen; HCV Ab = anti-hepatitis C; BMI = body mass index; eGFR, estimated glomerular filtration rate; statistical comparisons by

^a^ANOVA

^b^Chi squared

^c^Kruskal-Wallis

Of the 138 participants with DM, 38 (4.0% of the audit population) were newly diagnosed with DM. Their median HbA1c was 51 (inter-quartile range [IQR] 48–56) mmol/mol (6.8% [6.5–7.3]). Review of the clinical records indicated that 29 (76%) had been informed of their abnormal HbA1c and advised to see their General Practitioner (GP), in 23 the GP was notified while for eight no actions had been recorded.

**Figure 1.**
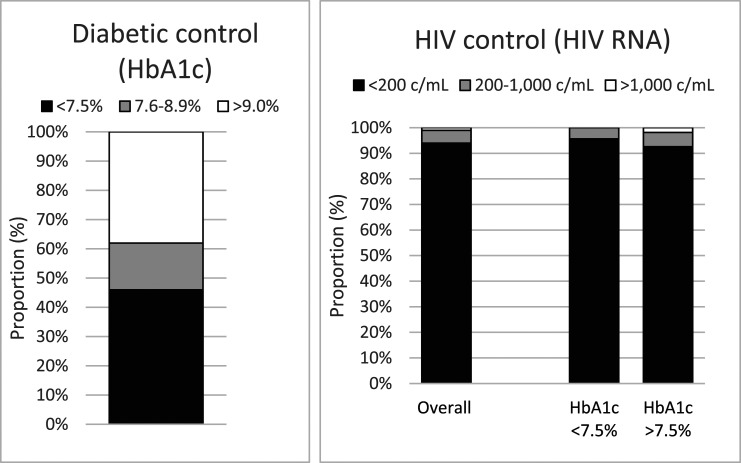
Diabetic and HIV control.

The 100 participants with a prior DM diagnosis had a median HbA1c of 62 (IQR 49–89) mmol/mol (7.8% [6.6–10.3]) and only 34% met the NICE target for glycaemia control and 46% the national audit standard. Notably, more than one third (38%) of participants had very poor glycaemia control (HbA1c ≥ 9.0% [75 mmol/mol]). This contrasts with excellent HIV control: 94%, 5% and 1%, respectively, had an HIV viral load <200, 200–1000 and >1000 copies/mL (Figure 1).

Of the 54 participants with an HbA1c of >7.5% (58 mmol/mol), 21 (39%) had their DM treatment intensified after the audit measurement, and 11 (20%) had either been seen by or referred to a diabetes specialist clinic. During follow up, six (11%) achieved glycaemia control (HbA1c ≤ 7.5%), seven (13%) were awaiting repeat HbA1c measurement, and 41 (76%) had persistently raised HbA1c measurements. Notably, most of these patients with poor glycaemia control also had higher than recommended blood pressures (which exceeded 140/90 in 57% and 160/100 in 21%) despite high levels of HIV control (93% had an undetectable HIV viral load, 6% had low level viraemia, and 2% had an HIV viral load >1000 copies/mL) (Figure 1).

## Discussion

Our audit indicates that inclusion of HbA1c in the annual laboratory order set allowed most people previously enrolled in the GEN-AFRICA study to be screened for DM despite pandemic disruptions to the HIV service. Our data indicate that there is a high burden of both undiagnosed and poorly controlled DM among ageing Black people with HIV in South London, and that poor DM control was frequently accompanied by sub-optimally or poorly controlled blood pressure. By contrast, HIV in this population was generally well controlled, with most participants having normal CD4 cell counts and suppressed HIV viral loads. Our data provide strong support for the inclusion of HbA1c in the laboratory monitoring of Black people with HIV and suggest additional measures may need to be provided to improve DM control and reduce health inequalities.

Early, intensively managed glycaemia control has sustainable long-term benefits in reducing microvascular and cardiovascular risk. Ethnic disparities in the management of T2DM continue to exist in the UK; compared to white people, those of Black ethnicity were shown to have poorer HbA1c, were less likely to be prescribed sodium-glucose cotransporter-2 inhibitors and glucagon-like peptide-1 agonists and to have annual testing for HbA1c and retinopathy.^8^ Failure to intensify treatment with noninsulin combination therapy or insulin within 12 months of an HbA1c >7.5% (therapeutic inertia) was also more common among Black individuals.^9^

As with antiretroviral therapy, knowledge and health beliefs have a major impact on black African and African Caribbean peoples’ adherence to their DM treatment; limited knowledge and understanding of DM and its risk factors, mistrust of the advice and treatments offered and a preference for natural treatments, fear of insulin, and a perception that diet- or tablet-controlled DM was a mild form that did not warrant serious concern have been reported in qualitative studies.^10,11^

We recently conducted focus groups discussions on long-term conditions with people of African ancestry with HIV in South London. These confirmed previously identified obstacles and additional challenges to the successful management of comorbidities such as T2DM including poor communication with and limited trust in non-HIV healthcare providers; self-stigma and concern about inadvertent disclosure of HIV status; difficulty in navigating the health care system; lack of peer support; financial and cultural barriers to implementing lifestyle interventions; and limited knowledge of and access to culturally-sensitive information about DM and its long complications.^12^

We conclude that T2DM and pre-diabetes are highly prevalent in Black people with HIV and that HbA1c testing, despite its limited sensitivity to detect T2DM in people with HIV, allows many of these to be newly diagnosed and DM control to be evaluated. We report a striking discrepancy between general good HIV control and poor DM control, and further studies are urgently required to explore the reasons for this so that effective programs can be put in place to prevent adverse health outcomes associated with poorly controlled T2DM. As Black populations are disproportionally affected by both HIV and DM, HIV services are well placed to diagnose DM, provide culturally sensitive information about DM, and participate in the management of DM.
